# Co-delivering a cGAS agonist and antigen via LNPs potentiates cancer immunotherapy

**DOI:** 10.1016/j.omtn.2025.102724

**Published:** 2025-10-10

**Authors:** Zhaoming Wu, Miao Zhang, Qianqian Ni

**Affiliations:** 1 Department of Diagnostic Radiology, Yong Loo Lin School of Medicine, National University of Singapore, Singapore 119074, Singapore; 2 Nanomedicine Translational Research Program, Yong Loo Lin School of Medicine, National University of Singapore, Singapore 117597, Singapore

## Main text

Therapeutic cancer vaccines hold great promise to transform oncology by inducing durable, antigen-specific immune responses. Yet, despite substantial technological advances, their clinical translation has been hindered by limited immunogenicity, suboptimal antigen presentation, and the immunosuppressive tumor microenvironments.^1^ In parallel, immune checkpoint blockade (ICB) has revolutionized cancer therapy, but its efficacy remains limited in “cold” tumors that lack sufficient cytotoxic T lymphocyte infiltration.^1^^,^^2^ Overcoming these challenges necessitates new combination strategies that not only promote antigen-specific antitumor responses but also provide robust innate immune activation to effectively prime T cell responses, circumvent immune suppression, and synergize ICB to enhance therapeutic efficacy.

A recent study demonstrates that an oligonucleotide-based cyclic GMP-AMP synthase (cGAS) agonist, Svg3, when co-delivered with peptide or mRNA vaccines in lipid nanoparticles (LNPs), potently activates type I interferon (IFN-I) responses, enhances antigen presentation, and substantially expands antigen-specific CD8^+^ T cells.^3^ In murine colorectal and human papillomavirus (HPV)-associated tumor models, Svg3-containing vaccines synergize with programmed cell death protein 1 (PD-1) blockade to elicit superior tumor control. These findings highlight cGAS-targeted adjuvants as a promising new direction in cancer vaccine design and underscore the translational potential of integrating upstream innate agonists with clinically validated LNP delivery systems. Nonetheless, they also raise important questions about dose optimization, translational safety, and the durability of responses in heterogeneous human tumors.

### Mechanistic innovation and platform advantages

**Figure 1:**
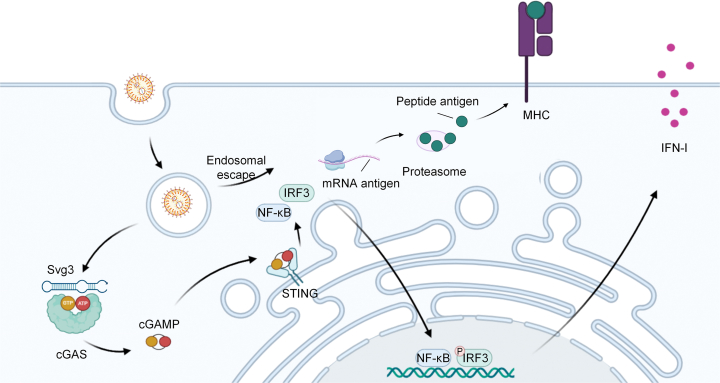
Intracellular process of tumor antigen-encoding mRNA and Svg3 co-delivered by LNPs Following endosomal escape, the loaded mRNA molecule is translated into the peptide antigen, which is subsequently processed—primarily by the proteasome—and presented on major histocompatibility complex class I (MHC-I) molecules, as well as on MHC-II molecules through cross-presentation pathways, enabling recognition by CD8^+^ and CD4^+^ T cells. In parallel, Svg3 activates the cGAS-stimulator of interferon genes (STING) axis, leading to cyclic GMP-AMP (cGAMP) production, STING activation, and downstream nuclear factor kappa-light-chain-enhancer of activated B cells/interferon regulatory factor 3 (NF-κB/IRF3) signaling, resulting in IFN-I secretion and pro-inflammatory cytokine release. Together, efficient antigen presentation and a STING-driven inflammatory, co-stimulatory microenvironment synergistically promote APC maturation and the robust priming of adaptive immune responses.

Svg3 activates the cGAS pathway, driving endogenous 2′3′-cGAMP production and sustained stimulator of interferon genes (STING) signaling. By engaging cGAS rather than directly targeting STING, Svg3 elicits amplified and prolonged IFN responses. In antigen-presenting cells (APCs), this activation enhances co-stimulatory molecule and chemokine expression, thereby improving T cell priming.^4^ Notably, when co-encapsulated in LNPs with peptide or mRNA antigens, Svg3 ensures synchronized delivery of antigen and innate stimuli—an essential feature to prevent tolerance and maximize immunogenicity (Figure 1). This strategy leverages the clinical maturity of LNP platforms, which have been well validated through mRNA vaccine development.^5^ LNPs enable efficient peptide/nucleic acid cargo encapsulation, promote lymphatic trafficking, and facilitate uptake by APCs, ensuring convergence of both antigen and adjuvant in draining lymph nodes. Compared with conventional adjuvants such as CpG (a Toll-like receptor 9 agonist) and other cyclic dinucleotide STING agonists,^6^ Svg3 offers both mechanistic novelty and practical compatibility with nucleic acid vaccine technologies.

### Translational promise and preclinical efficacy

**Figure 2:**
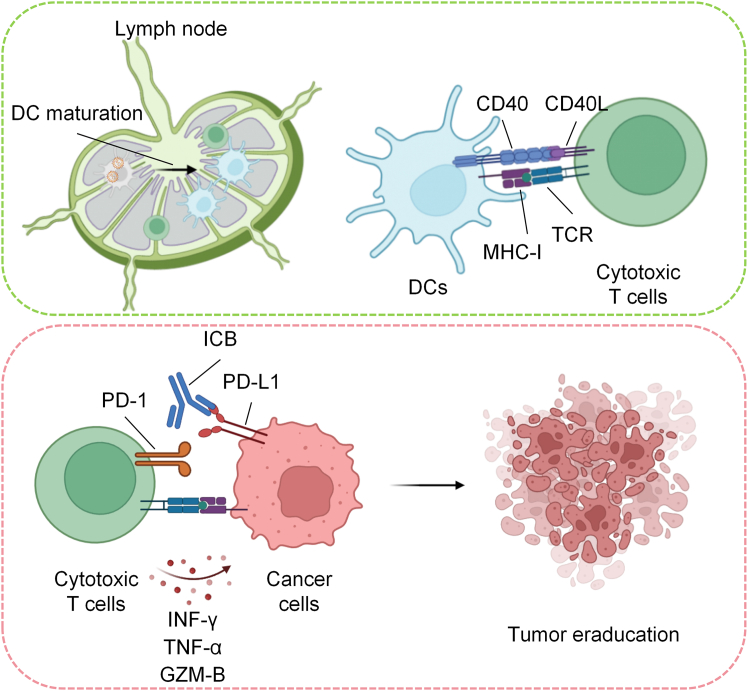
Activated APCs prime CD8^+^ T cells in lymph nodes to mediate tumor eradication Mature APCs migrate to draining lymph nodes and present antigenic peptides via MHC-I to naive CD8^+^ T cells while providing co-stimulatory signals and cytokines. This primes expansion and differentiation of cytotoxic T lymphocytes (CTLs) that home to the tumor and kill cancer cells via interferon-gamma (IFN-γ), tumor necrosis factor-alpha (TNF-α), and granzyme B (GZM-B). Tumor-expressed PD-L1 can suppress CTL function through PD-1 engagement, while ICB targeting PD-1/PD-L1 restores T cell effector activity. Svg3-induced STING activation, together with mRNA-driven antigen expression, potentiates APC priming and enhances the magnitude and quality of the antitumor T cell response.

The preclinical findings of this co-delivery strategy are compelling. In MC38 colorectal cancer and TC-1 HPV-associated tumor models, Svg3-antigen vaccines markedly increased the magnitude of antigen-specific CD8^+^ T cells, often 2- to 4-fold compared with antigen-alone formulations. These T cells exhibited effector and memory phenotypes, supporting both acute tumor clearance and potential long-term protection. Notably, Svg3 also induced PD-1 expression on antigen-specific T cells, establishing a clear biological rationale for combination with PD-1 blockade. Indeed, the combination of Svg3 vaccines with anti-PD-1 therapy significantly enhanced tumor control. In TC-1 tumor-bearing mice, durable complete regression was achieved in 2 of 7 tumors, while either antigen or Svg3 alone has no effect in tumor regression. These data suggest that Svg3 can convert poorly immunogenic tumors into ICB-responsive settings, thereby addressing the concerns of limited response rates of conventional ICB therapy (Figure 2).

### Challenges and open questions

Despite the promise, several issues temper enthusiasm and warrant deeper exploration before clinical translation. First, administration dose is a major concern of this co-delivery strategy. High dose of Svg3 has been shown to suppress mRNA translation, revealing a narrow therapeutic window. Achieving the right balance between adjuvanticity and antigen expression will be crucial for maximizing efficacy while ensuring safety.^7^ In addition, systemic IFN-I induction presents potential risks. Although beneficial for promoting immunity, excessive IFN signaling may have neurotoxic potential.^8^ Therefore, thorough evaluation of biodistribution, pharmacokinetics, and cytokine profiles in large animal models will be indispensable. Murine tumor models, though informative, often overpredict efficacy relative to human cancers, which are more heterogeneous and evolve complex immune evasion mechanisms.^9^^,^^10^ It remains uncertain whether Svg3 vaccines can effectively overcome challenges such as antigen loss, immunosuppressive myeloid populations, and stromal barriers in human cancers. Lastly, the durability of immune responses is a critical clinical consideration. Systematic assessment is needed to determine whether Svg3-based vaccines can generate long-lasting memory T cells that persist beyond transient IFN activation.

### Outlook

This study establishes a conceptual foundation for incorporating cGAS agonists into nanovaccines. Beyond oncology, Svg3 or related molecules could also be explored as adjuvants in infectious disease vaccines, where strong IFN-driven immunity is advantageous. Within cancer, pairing Svg3 vaccines with personalized neoantigen discovery pipelines could create tailored immunotherapies, expanding the scope of precision medicine. Future work could prioritize three directions: (1) systematic dose and formulation optimization to balance translation efficiency and innate activation, (2) rigorous preclinical toxicology to delineate safety margins, and (3) clinical exploration in cancers with poor responsive rates to ICB, such as microsatellite-stable colorectal cancer or pancreatic cancer. The modularity of LNP platforms further facilitates rapid iteration and adaptation to diverse antigen contexts.

In summary, co-delivery of a cGAS agonist and tumor antigens in LNPs constitutes an innovative and potent immunotherapy strategy, integrating upstream innate activation with robust antigen presentation. By synergizing with PD-1 blockade, this approach could help broaden the clinical benefit of cancer immunotherapy. However, several translation challenges including optimal dosing, safety considerations, and the inherent cancer heterogeneity and complexity of cancers must be addressed. With meticulous development, Svg3-based nanovaccines could significantly enhance the immuno-oncology toolkit and transform the way vaccines and checkpoint blockade are integrated into clinical practice.

## Declaration of interests

The authors declare no competing interests.
